# Petrolatum-based ointment application induces swelling of the PRESERFLO Microshunt

**DOI:** 10.1007/s00417-025-07075-2

**Published:** 2026-01-13

**Authors:** Ryo Tomita, Taiga Inooka, Takato Kajita, Hideyuki Shimizu, Ayana Suzumura, Jun Takeuchi, Tsuyoshi Matsuno, Hidekazu Inami, Koji M. Nishiguchi, Atsushi Noro, Kenya Yuki

**Affiliations:** 1 Department of Ophthalmology, Nagoya University Graduate School of Medicine, 65 Tsurumai-cho, Showa-ku, Nagoya, 466-8550 Aichi Japan; 2 Department of Molecular & Macromolecular Chemistry, Graduate School of Engineering, Nagoya University, Nagoya, 464-8603 Aichi Japan; 3 Institute of Materials Innovation, Institutes of Innovation for Future Society, Nagoya University, Nagoya, 464-8601 Aichi Japan; 4 Research Center for Net-Zero Carbon Society, Institutes of Innovation for Future Society, Nagoya University, Nagoya, 464-8601 Aichi Japan

**Keywords:** Glaucoma, PRESERFLO microshunt, Bleb leak, Ophthalmic ointment

## Abstract

**Purpose:**

To report the clinical cases of PRESERFLO MicroShunt swelling following exposure to petrolatum-based ophthalmic ointment and to experimentally identify the cause and composition of the swollen MicroShunt.

**Methods:**

A retrospective case series and an *in vitro* experimental study were conducted. The clinical series included three glaucoma patients with swollen MicroShunts after ointment exposure and four patients whose explanted MicroShunts showed no swelling without ointment exposure. *In vitro* experiments were performed using unused MicroShunts incubated in 0.3% ofloxacin ophthalmic ointment. A time-course immersion study was conducted for 24 h at room temperature, with microscopy documenting dimensional changes. After 24 h and 3 months, swollen devices underwent quantitative analysis using proton nuclear magnetic resonance (^1^H-NMR) spectroscopy to determine the relative weight composition of polystyrene-block-polyisobutylene-block-polystyrene (SIBS) and absorbed ointment.

**Results:**

All three MicroShunts exposed to ointment exhibited marked swelling, whereas those without exposure showed no swelling. *In vitro*, ointment-exposed devices displayed similar swelling, became friable, and fractured after 24 h. Dimensional analysis demonstrated progressive increases, with outer diameter expanding 1.44-fold and fin width 1.29-fold by 24 h. ^1^H-NMR analysis showed that swollen devices consisted of approximately 55% SIBS and 45% ointment by weight after 24 h, progressing to 27% SIBS and 73% ointment after 3 months.

**Conclusions:**

Direct contact between petrolatum-based ophthalmic ointment and the SIBS-based MicroShunt induced markedly, time-dependent swelling that compromises the its structural integrity. Thus, the use of ophthalmic ointment should be avoided in patients with an exposed MicroShunt.

**Supplementary information:**

The online version contains supplementary material available at 10.1007/s00417-025-07075-2.

## Key Messages

*****What is known***:**

- The PRESERFLO MicroShunt, composed of the biocompatible polymer polystyrene-block-polyisobutylene-block-polystyrene (SIBS), is a novel filtration device used to lower intraocular pressure in glaucoma, showing excellent biocompatibility and a low tendency to induce inflammation and scarring.

*****What is new***:**

- We report the first clinical and experimental evidence of MicroShunt swelling following exposure to petrolatum-based ophthalmic ointment.
- Direct contact of ophthalmic ointment with the MicroShunt causes swelling that can compromise device integrity, as demonstrated both clinically and *in vitro*.
- Ophthalmic ointment should not be used when the MicroShunt is exposed, and its use may be better avoided when conjunctival integrity is compromised or a bleb leak is present.

**Supplementary information:**

The online version contains supplementary material available at 10.1007/s00417-025-07075-2.

## Introduction

Glaucoma is a leading cause of blindness [1, 2]. To surgically lower intraocular pressure (IOP) and prevent glaucoma progression, the PRESERFLO MicroShunt (Santen Inc., Osaka, Japan) is used as a novel filtration device to treat patients with progressive glaucoma as an alternative to trabeculectomy [3]. The MicroShunt, a device measuring 8.5 mm in length with a 70 μm lumen, comprises polystyrene-block-polyisobutylene-block-polystyrene (SIBS), an ABA-type triblock copolymer–based thermoplastic elastomer [4, 5] with excellent biocompatibility and a lower tendency than silicone to induce inflammation, capsular tissue formation, collagen deposition, and myofibroblast differentiation [6–8]. 

However, SIBS is both hydrophobic and lipophilic and can totally dissolve in non-polar solvents such as toluene and hexanes [9, 10]. It can also dissolve or swell upon contact with petrolatum, a synthetic semisolid mixture of hydrocarbons obtained from petroleum and widely used as a base in ophthalmic ointments. Thus, contact between the MicroShunt and ophthalmic ointment could potentially lead to lipid uptake and swelling of the MicroShunt [9]. However, this has not been studied previously. Although the Instructions For Use (IFU) of the PRESERFLO MicroShunt states, under the heading of WARNINGS, that “The PRESERFLO MicroShunt should not be subjected to direct contact with petrolatum-based (i.e., petroleum jelly) materials (e.g., ointments, dispersions, etc.).” we believe it is important to publish our findings as many surgeons may not fully review the IFU [11]. Here, we report three cases in which MicroShunt swelling was observed when ophthalmic ointment was applied to the exposed MicroShunt. Additionally, because we hypothesized that the ophthalmic ointment was causally implicated, we incubated unused MicroShunt in the ophthalmic ointment *in vitro* to further demonstrate swelling.

## Methods

Three cases of MicroShunt swelling and four cases without MicroShunt swelling but with MicroShunt removal during secondary glaucoma surgery after MicroShunt implantation were identified at Nagoya University Hospital between May 2024 and June 2025, and *in vitro* experiment 1, which evaluated ophthalmic ointment-induced swelling, was conducted in the laboratory of the Department of Ophthalmology, Nagoya University Graduate School of Medicine. *In vitro* experiment 2 was conducted in the Laboratory of Physical Chemistry of Macromolecules, Department of Molecular & Macromolecular Chemistry Graduate School of Engineering, Nagoya University. According to the Institutional Review Board of Nagoya University Hospital, this deidentified case series was exempt from ethical review. Informed consent was obtained from all patients.

For *in vitro* experiment 1, unused MicroShunt was completely coated with 0.3% ofloxacin ophthalmic ointment containing petrolatum as its base (Tarivid^®^, Santen Inc., Osaka, Japan) and incubated at 37 °C for 7 days.

In *in vitro* experiment 2, designed to characterize swelling dynamics at higher temporal resolution, each unused MicroShunt was completely submerged in 0.3% ofloxacin ophthalmic ointment at room temperature. Continuous video microscopy was conducted during the first 3 h of immersion to capture real-time changes. At cumulative immersion intervals of 3, 6, 9, 12, and 24 h, the MicroShunt was retrieved, gently wiped with a laboratory tissue to remove excess ophthalmic ointment, and immediately documented via optical microscopy using a BX51 optical microscope (Olympus, Tokyo, Japan) and gross-appearance imaging to monitor progressive dimensional enlargement and surface morphology. After each imaging session, the MicroShunt was returned to its ointment bath for further incubation. Following the 24-hour and 3-month time points, the samples were carefully and thoroughly wiped with a laboratory tissue to minimize any residual ophthalmic ointment on the surface, and analyzed using proton nuclear magnetic resonance (^1^H-NMR) spectroscopy, which uses the principle of nuclear magnetic resonance (NMR) to characterize the chemical environment of hydrogen atoms as well as the chemical structure and composition of organic compounds or target molecules. In this way, the relative weight of the SIBS polymer matrix and ophthalmic ointment components were estimated. All ^1^H-NMR spectra were measured using an AVANCE III HD 500 MHz spectrometer (Bruker, Billerica, MA, USA) with deuterated chloroform (CDCl₃) as the solvent. The relative weight composition of SIBS and the absorbed 0.3% ofloxacin ophthalmic ointment was determined using two independent analytical methods. In both methods, the intrinsic weight ratios of styrene and isobutylene blocks in pure SIBS polymer were first determined as reference values. The first method involved mixing a known weight of pure polystyrene with ophthalmic ointment in a 1:1 weight ratio to create a calibration standard that established the integral values of NMR proton signals and the mass conversion factor. This factor was used to determine the composition of the swollen MicroShunt from the ^1^H-NMR spectrum. The second independent method modeled the main component of the ophthalmic ointment using its average molecular formula. This assumption was derived from the proton ratios of the pure ophthalmic ointment spectrum. This model enabled direct conversion of the proton integral ratios of the SIBS and the ointment into weight ratios using their respective molecular weights.

## Results

### Case 1

**Fig. 1:**
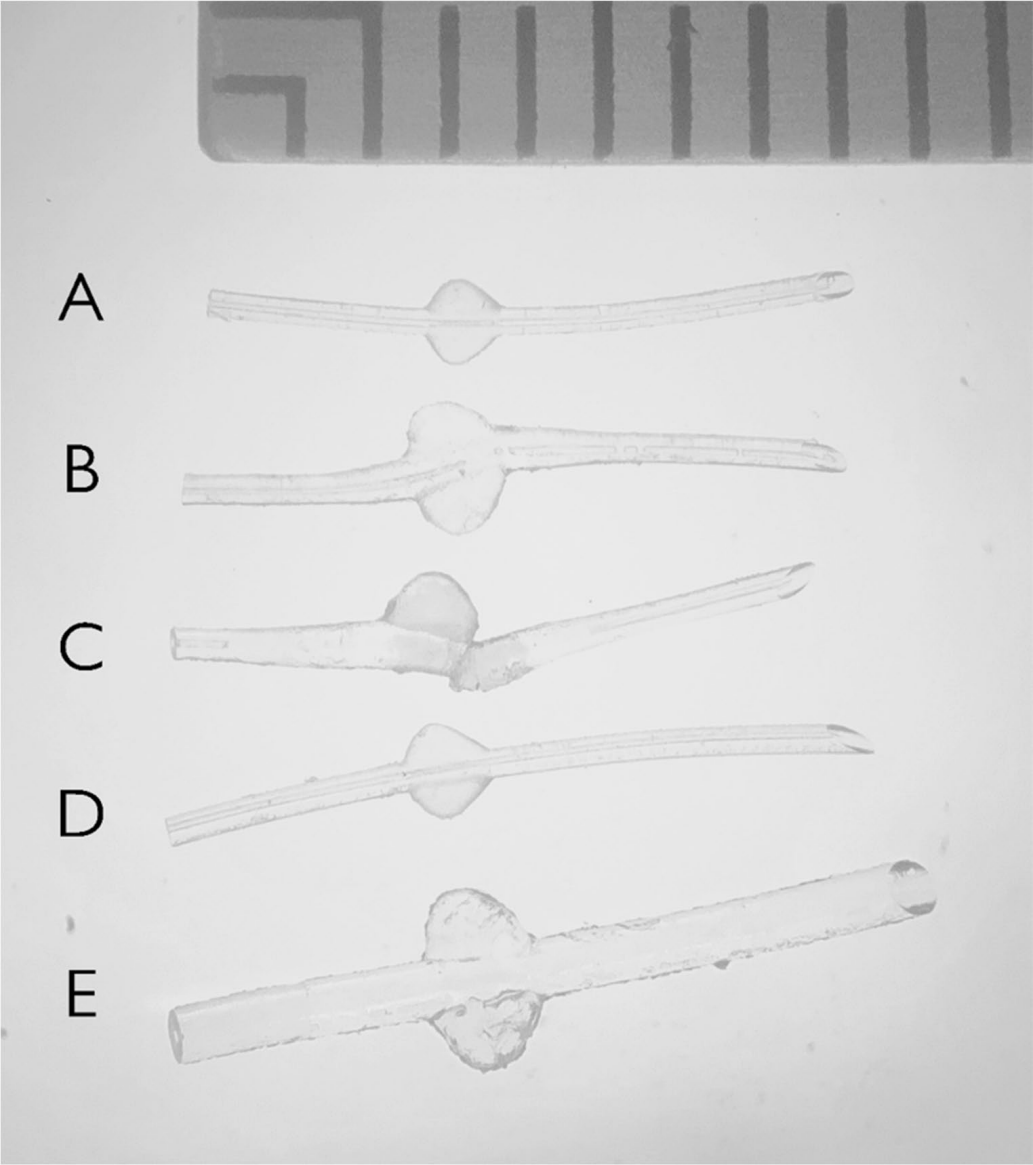
Comparative photographs of MicroShunts. Photographic comparison of MicroShunt illustrating size changes. (**A**) Unused MicroShunt (control). (**B**) MicroShunt explanted from case 1, showing localized swelling around the fin. (**C**) MicroShunt explanted from case 2, exhibiting diffuse swelling with fracture and loss of one fin. (**D**) MicroShunt removed during secondary surgery without a bleb leak, MicroShunt exposure, or use of ophthalmic ointment. (**E**) MicroShunt that markedly swelled after *in vitro* exposure to ophthalmic ointment for one week

**Fig. 2:**
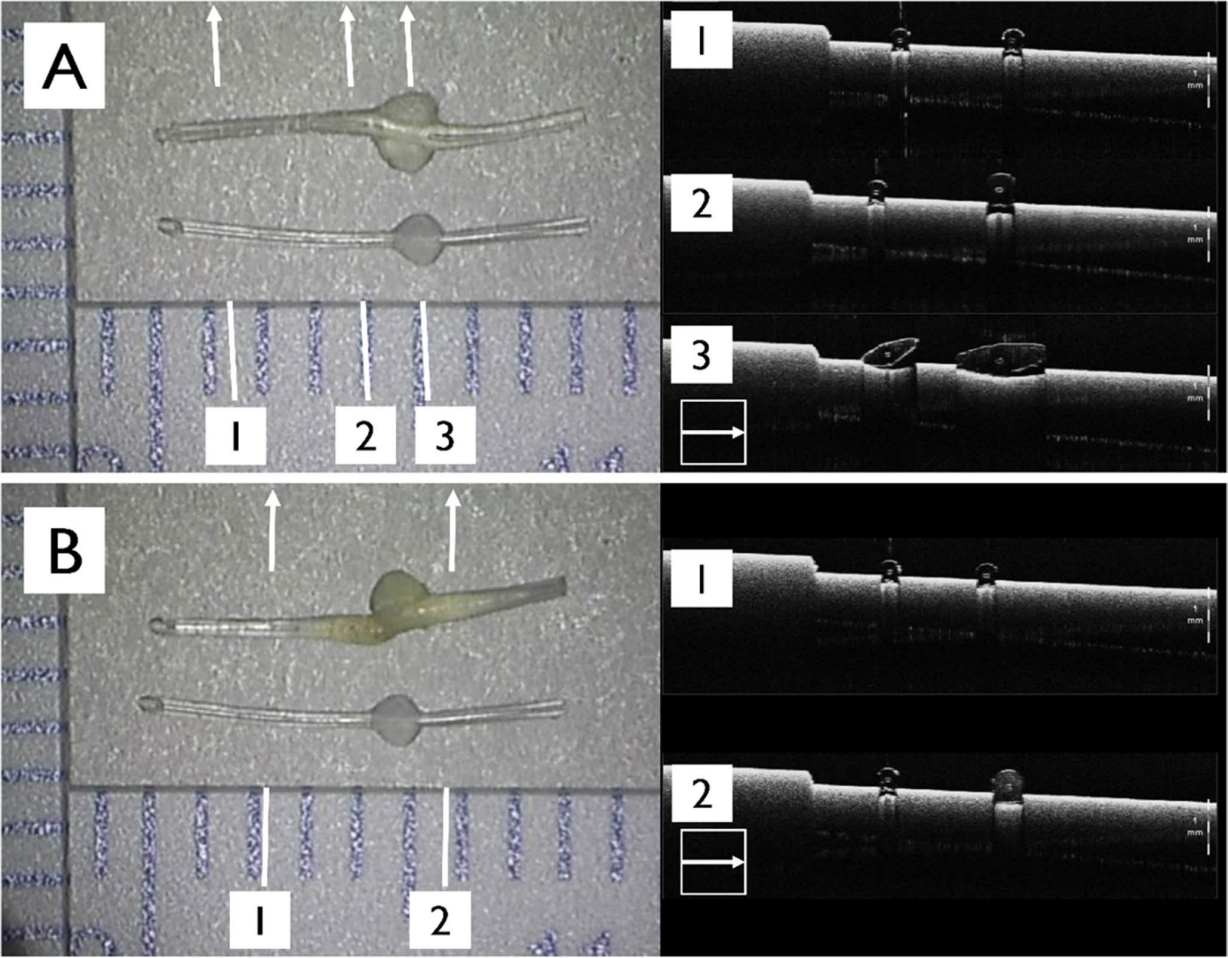
Optical coherence tomography (OCT) images of swollen MicroShunts. (**A**) OCT image of a MicroShunt explanted from case 1 (top) and an unused MicroShunt (bottom). The distal portion that had been positioned intraocularly did not swell (line 1), whereas swelling was evident near the fins, the exposed part (line 2 and 3). The luminal structure was preserved throughout. (**B**) OCT image of a MicroShunt explanted from case 2 (top) and an unused MicroShunt (bottom). The intraocular distal portion did not swell (line 1), whereas swelling was observed near the fins (line 2). The MicroShunt was fractured near the fin, and the lumen could not be reliably assessed by OCT

A 54-year-old male with atopic dermatitis and a remote history of childhood cataract extraction underwent MicroShunt implantation in the right eye for secondary open-angle glaucoma at the referring clinic. 0.1% dexamethasone ophthalmic ointment (Nitto Medic Co., Ltd., Saga, Japan) was applied for 1 month postoperatively. Needling revision was required at 6 months for early encapsulation. At 1 year after the initial surgery, progressive IOP elevation necessitated a bleb revision with explantation of the original MicroShunt and replacement with a new MicroShunt due to a fracture of the MicroShunt that had previously been implanted. Despite maximal medical therapy, IOP remained at 45 mm Hg. The patient was then referred to our hospital, and micropulse transscleral cyclophotocoagulation was performed based on the patient’s preference. Rupture of the conjunctiva of the bleb and MicroShunt exposure were observed nine weeks after the bleb revision at which point 0.3% ofloxacin ophthalmic ointment was initiated. Five days later, the MicroShunt was explanted, and an Ahmed glaucoma valve was implanted in an uninvolved quadrant. The explanted MicroShunt was swollen compared to an unused MicroShunt (Fig. 1A and B). Optical coherence tomography confirmed MicroShunt swelling and patency of the lumen (Fig. 2A). Postoperative IOP remained in the low teens with no IOP-lowering agents during a follow-up at 3 months.

## Case 2

A 51-year-old male with a complex ocular history, including prior scleral buckle, cataract extraction, ab externo trabeculotomy, and intraocular lens refixation, underwent MicroShunt implantation in the left eye at a referring clinic, and required needling revision after 5 weeks. Postoperatively, 0.1% dexamethasone ophthalmic ointment was administered once daily for a total of 9 weeks, starting from the day of the first MicroShunt implantation until 4 weeks after needling. However, the IOP had risen to 60 mm Hg by week 14 after first MicroShunt implantation. The referring clinic’s records did not document any exposure related to the MicroShunt or bleb leak. Then, the patient was referred to our hospital and MicroShunt exposure from the conjunctiva was evident without bleb leak. On the next day, the MicroShunt was removed, and an EXPRESS glaucoma filtration device (Alcon Inc., Fort Worth, TX, USA) was inserted into an uninvolved quadrant. The explanted MicroShunt was swollen and friable. During removal, it broke and one fin detached (Figs. 1C and 2B). Postoperative IOP remained in the low teens without the use of IOP-lowering agents at the 2-month follow-up.

## Case 3

**Fig. 3:**
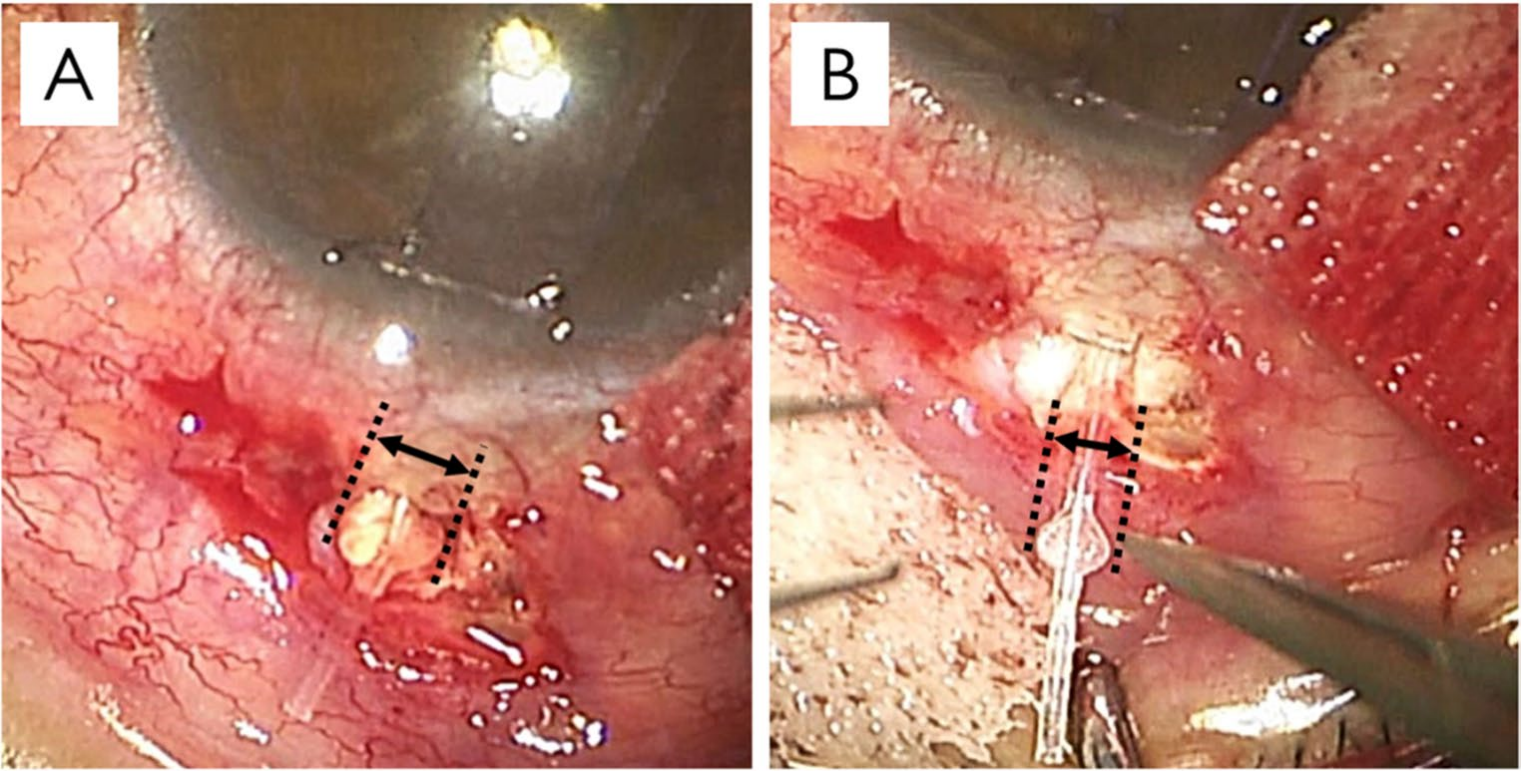
Swollen MicroShunt in case 3. Images captured from the recorded movie of the surgery showed MicroShunt swelling (**A**) and the new unused MicroShunt (**B**)

A 59-year-old female with primary open-angle glaucoma in the left eye underwent subconjunctival MicroShunt implantation after an IOP of 33 mm Hg proved refractory to maximal medical therapy. A Seidel-positive bleb leak appeared 2 weeks postoperatively and was closed with conjunctival suturing, yet low-grade bleb leak persisted, and 0.3% ofloxacin ophthalmic ointment was initiated twice daily. Partial MicroShunt exposure was noted at 5 weeks after surgery and the following bleb revision revealed marked swelling of the MicroShunt at 6 weeks (Fig. 3A). The explanted MicroShunt, which fractured when a fin detached upon being grasped, was discarded and replaced with a new MicroShunt (Fig. 3B) that was covered with conjunctiva. Two weeks later, re-exposure recurred. Therefore, the MicroShunt was removed, and a trabeculectomy was performed in an uninvolved quadrant. IOP subsequently stabilized between 7 and 9 mm Hg without medication at the latest follow-up (10 months).

## Case 4

An 84-year-old female with pseudoexfoliation glaucoma in the right eye underwent subconjunctival MicroShunt implantation. No bleb leak or rupture occurred after the implantation, and no ophthalmic ointment was used. Due to increased IOP, Ahmed glaucoma valve implantation was performed 6 months after the initial procedure, and the MicroShunt was removed. The dimensions of the explanted MicroShunt matched those of an unused MicroShunt (Fig. 1D**)**.

### Additional cases

Three additional cases also required MicroShunt explantation and subsequent glaucoma surgery due to uncontrolled IOP. The three explanted MicroShunts from these cases revealed no evidence of swelling (see Online Resource 1). Notably, one case involved device exposure that was repaired four times with conjunctival suturing but without the use of any ophthalmic ointment. Table 1 summarizes all the clinical cases.

**Table 1:** Characteristics of patients who underwent microshunt removal

Case no.	Age (y)	Sex	Diagnosis	Follow-up after MicroShunt implantation (months)	Secondary surgery	Bleb leak	Exposure of MicroShunt	Use of ophthalmic ointment	MicroShunt swelling
1	54	Male	Secondary open-angle glaucoma to atopic cataract surgery	14	Ahmed glaucoma valve	-	+	0.1% dexamethasone, 0.3% Ofloxacin	+
2	51	Male	Secondary open-angle glaucoma to complex history of intra ocular surgery	3	EX-PRESS Glaucoma Filtration Device	-	+	0.1% dexamethasone	+
3	59	Female	Primary open-angle glaucoma	2	Trabeculectomy	+	+	0.3% ofloxacin	+
4	84	Female	Pseudoexfoliation glaucoma	6	Ahmed glaucoma valve	-	-	-	-
5	57	Male	Secondary glaucoma to cytomegalovirus corneal endotheliitis	2	Trabeculectomy	-	-	-	-
6	63	Male	Secondary glaucoma to cytomegalovirus corneal endotheliitis	4	Ahmed glaucoma valve	-	-	-	-
7	67	Female	Pseudoexfoliation glaucoma	6	Ahmed glaucoma valve	+	+	-	-

### *In vitro* experiment 1

Incubation of an unused MicroShunt with 0.3% ofloxacin ophthalmic ointment resulted in marked, diffuse swelling that was morphologically similar to the explanted MicroShunts in the clinical cases (Fig. 1E).

### *In vitro* experiment 2

**Fig. 4:**
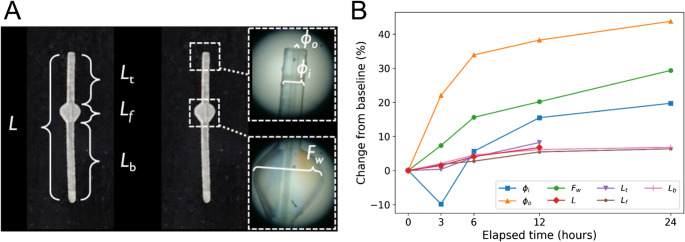
Time-dependent dimensional changes of the MicroShunt during *in vitro *immersion in ophthalmic ointment. (**A**) A schematic illustrates the key dimensions of the MicroShunt that were measured: internal lumen diameter (*φ*_*i*_), outside diameter (*φ*_*o*_), fin width (*F*_*w*_), total length (*L*), length of the top side (*L*_*t*_), length of the fins (*L*_*f*_), length of the bottom side (*L*_*b*_), and length of the bottom side (*L*_*b*_). (**B**) The graph plots the percentage change from baseline for these dimensions: internal lumen diameter (*φ*_*i*_, blue squares), outside diameter (*φ*_*o*_, orange triangles), fin width (*F*_*w*_, green circles), total length (*L*, red diamonds), length of the top side (*L*_*t*_, brown circle), length of the fins (*L*_*f*_, purple inverted triangle), and length of the bottom side (*L*_*b*_, pink vertical line marker). All external dimensions grew over the 24-hour period. Measurements of total length (*L*) and length of the top side (*L*_*t*_) were discontinued after 12 h because the device fractured due to material weakening. Absolute measurement values for each time point are provided in Online Resource 2

In *in vitro* experiment 2, all dimensions of the MicroShunt increased progressively with ophthalmic ointment exposure (Fig. 4). Relative to baseline, the outside diameter (*φ*_*o*_) expanded from 0.345 mm to 0.496 mm at 24 h (a 1.44-fold increase), and the fin width (*F*_*w*_) grew from 1.09 mm to 1.41 mm (a 1.29‐fold increase). Likewise, the total MicroShunt length (*L*) extended from 8.30 mm at time zero to 8.86 mm by 12 h (a 1.07‐fold increase). Beyond 12 h, the MicroShunt fractured and could not be measured. The internal lumen diameter (*φ*_*i*_) exhibited an initial constriction, declining from 0.071 mm at baseline to 0.064 mm after 3 h, before returning to and ultimately exceeding the original diameter (0.075 mm at 6 h, 0.082 mm at 12 h). The dimensions at each time point are shown in Online Resource 2. These dynamic changes in the exterior and interior dimensions indicate that direct immersion in petrolatum‐based ophthalmic ointment induces significant, time‐dependent swelling of the SIBS polymer matrix. The kinetics of the dimensional change over the first 3 h are shown in Online Resource 3. ^1^H-NMR analysis of the pure MicroShunt polymer (i.e., SIBS) revealed its composition to be 18% styrene and 82% isobutylene by weight. The spectrum of pure ophthalmic ointment consisted almost exclusively of peaks derived from -CH₃, -CH₂-, and > CH-, indicating that the main component was a mixture of long-chain alkanes. The ^1^H-NMR spectrum of the swollen MicroShunt showed clear signals from the SIBS polymer and the absorbed ophthalmic ointment. The two quantitative methods for the swollen MicroShunt, which were performed because it was unclear which type of alkane the ophthalmic ointment contained, were consistent with each other. The first method (using a polystyrene calibration standard) found that the composition of the swollen MicroShunt was 55% SIBS and 45% ophthalmic ointment by weight (Weight of SIBS: Weight of ophthalmic ointment = 1:0.805) after 24 h immersion and 27% SIBS and 73% ophthalmic ointment by weight (Weight of SIBS: Weight of ophthalmic ointment = 1:2.69) after 3 months. The second method (assuming a representative structure of the ointment [C_23.6_H_48.6_]) yielded almost identical compositions (SIBS 56% and ophthalmic ointment 44% by weight after 24 h, and SIBS 28% and ophthalmic ointment 72%). Details of these analyses are provided in Online Resource 4.

## Discussion

Compared with trabeculectomy, implantation of a MicroShunt has been associated with a lower risk of postoperative hypotony and reduced need for subsequent surgical interventions [12, 13]. Although several case reports have documented MicroShunt exposure [14, 15], to our knowledge, neither clinical case reports of MicroShunt swelling nor experimental evidence of ointment-induced swelling have been published. This case series, together with the *in vitro* experiments, showed that physical contact with an ophthalmic ointment causes the MicroShunt polymer to significantly swell.

SIBS is used for implantable devices or biocompatible coatings because SIBS causes negligible foreign body reaction [9, 16, 17]. However, SIBS is lipophilic. Thus, when it comes into contact with petrolatum, the oil molecules penetrate between the polymer chains and cause swelling [9, 10]. The manufacturer’s instructions explicitly advise against direct exposure of the implant to ophthalmic ointment [11]. In cases 1 and 3, ophthalmic ointment was used even after MicroShunt exposure. In cases 2 and 3, ophthalmic ointment was initiated before MicroShunt exposure was recognized. In case 2, when the patient first visited our hospital, the MicroShunt was already exposed, and the use of ophthalmic ointment was discontinued. In case 3, a bleb leak was observed 2 weeks postoperatively. Subsequently, ophthalmic ointment was initiated, and partial MicroShunt exposure was first noted at 5 weeks followed by marked MicroShunt swelling during revision surgery at 6 weeks. Consequently, the temporal relationship between the use of ophthalmic ointment and the exposure of the MicroShunt is unclear in cases 2 and 3. However, the critical role of ophthalmic ointment is underscored by the cases in which it was not used. In these cases, the explanted MicroShunts were not swollen. Notably, one case developed MicroShunt exposure but was managed without ophthalmic ointment. In this case, the MicroShunt was not swollen. This suggests that direct contact with the ophthalmic ointment, rather than MicroShunt exposure itself, causes the MicroShunt to swell. Whether the use of ophthalmic ointment should be contraindicated in eyes with a bleb leak but without MicroShunt exposure warrants further investigation. Nevertheless, our findings indicate that ophthalmic ointment should be discontinued immediately after MicroShunt exposure is documented, and it may also be better to discontinue the use of ophthalmic ointment if bleb leak exists.

*In vitro* experiments demonstrated time-dependent swelling of the MicroShunt upon continuous ophthalmic ointment exposure, with its outside diameter and fin width increasing steadily over 24 h. Although progressive lumen constriction was anticipated as the polymer imbibed ophthalmic ointment, the internal lumen diameter narrowed transiently after 3 h before subsequently increasing. While the cause for this initial constriction is unknown, the eventual increase in diameter mirrored the preserved luminal patency observed in explanted devices from our clinical cases. Although luminal patency was maintained, the clinical consequences of swelling on filtration function remain uncertain.

In this study, ^1^H-NMR spectroscopy was used to quantitatively characterize the expansion of a MicroShunt based on SIBS when exposed to 0.3% ofloxacin ophthalmic ointment. After 24 h of immersion, the MicroShunt was composed of approximately 55% polymer and 45% absorbed ointment by weight. After 3 months, the composition changed to 27% polymer and 73% ointment. The results of the two independent quantification methods agreed with each other. To accurately determine these compositional changes, we employed ^1^H-NMR rather than simple gravimetric analysis because the MicroShunt is extremely lightweight, making it difficult to detect minute weight changes. Furthermore, completely removing the surface ointment physically without affecting the absorbed ointment is technically challenging. ^1^H-NMR allows for the precise determination of the compositional ratio of the polymer to the ointment, providing a more accurate assessment of swelling.

Ultimately, the significant degree of swelling may have clinically important implications. Contact with ophthalmic ointment may significantly alter the physical dimensions and mechanical properties (e.g., rigidity and flexibility) of MicroShunts, including hydrodynamic resistance. Therefore, further investigation is required to assess the impact of this swelling on MicroShunt functionality. As indicated in the manufacturer’s instructions advising against exposure of the implant to ophthalmic ointment, our findings demonstrate that such conditions readily induce swelling of the SIBS polymer and therefore clinicians should be aware of this potential risk. In fact, by 24 h the polymer weakened to an extent that the implant fractured, rendering total length measurement impossible. A comparable MicroShunt rupture was observed in cases 2 and 3, suggesting that ophthalmic ointment immersion may compromise MicroShunt structural integrity. Future device development should prioritize materials that remain chemically and physically stable when in contact with ophthalmic ointments. Alternatively, the use of water-based (hydrophilic) ointments may avoid swelling of the SIBS polymer, allowing the continued safe use of the current SIBS-based MicroShunt. However, we have not studied this interaction at this time.

Our study has several limitations. In cases 2 and 3, the temporal relationship between bleb leak and ophthalmic ointment use was unclear, and it is unknown whether ophthalmic ointment use affected the occurrence of exposure. Furthermore, it remains unknown whether swelling affects filtration function. However, the result of *in vitro* experiment 2 showed that the lumen was maintained.

In conclusion, direct contact of ophthalmic ointment with the MicroShunt causes MicroShunt swelling. The clinical consequences of this swelling on filtration function remain unclear. However, swelling can compromise device integrity, as demonstrated by fracture observed clinically and *in vitro.* Therefore, ophthalmic ointment should not be used in cases where MicroShunt is exposed. It may also be better to avoid ophthalmic ointment use when conjunctival integrity is compromised and bleb leak is present.

## Supplementary information

Below is the link to the electronic supplementary material.

Supplementary File 1 (PDF 162 KB)

Supplementary File 2 (PDF 339 KB)

Supplementary File 3 (PDF 575 KB)

Supplementary File 4 (mp4 9.51 MB)

## Data Availability

The datasets of the current study are not publicly available due to institutional data protection policies but are available from the corresponding author on reasonable request.
