# Supplementary material for: Petrolatum-based ointment application induces swelling of the PRESERFLO Microshunt
Source: Graefes Arch Clin Exp Ophthalmol. 2026 Jan 13;264(5):1489–97. doi: 10.1007/s00417-025-07075-2 (PMC13091893; doi:10.1007/s00417-025-07075-2)
Supplement: Supplementary file 1 — (PDF 162 KB) [file 417_2025_7075_MOESM1_ESM.pdf]

Online Resource 2. Dimensional Changes of MicroShunt After Immersion in ophthalmic ointment Over Time.

Time (h)	0	3	6	12	24
φ_i (mm)	0.071	0.064	0.075	0.082	0.085
φ_o (mm)	0.345	0.421	0.462	0.477	0.496
F_w (mm)	1.09	1.17	1.26	1.31	1.41
L (mm)	8.30	8.42	8.64	8.86	-
L_t (mm)	2.80	2.81	2.91	3.03	-
L_f (mm)	1.10	1.12	1.13	1.16	1.17
L_b (mm)	4.40	4.49	4.60	4.67	4.70

φ_i: internal lumen diameter, φ_o: outside diameter, F_w: width of the fin, L: total length, L_t: length of the top side, L_f: length of the fin, L_b: length of the bottom side, -: data not available.
