# Supplementary material for: Petrolatum-based ointment application induces swelling of the PRESERFLO Microshunt
Source: Graefes Arch Clin Exp Ophthalmol. 2026 Jan 13;264(5):1489–97. doi: 10.1007/s00417-025-07075-2 (PMC13091893; doi:10.1007/s00417-025-07075-2)
Supplement: Supplementary file 2 — (PDF 339 KB) [file 417_2025_7075_MOESM2_ESM.pdf]

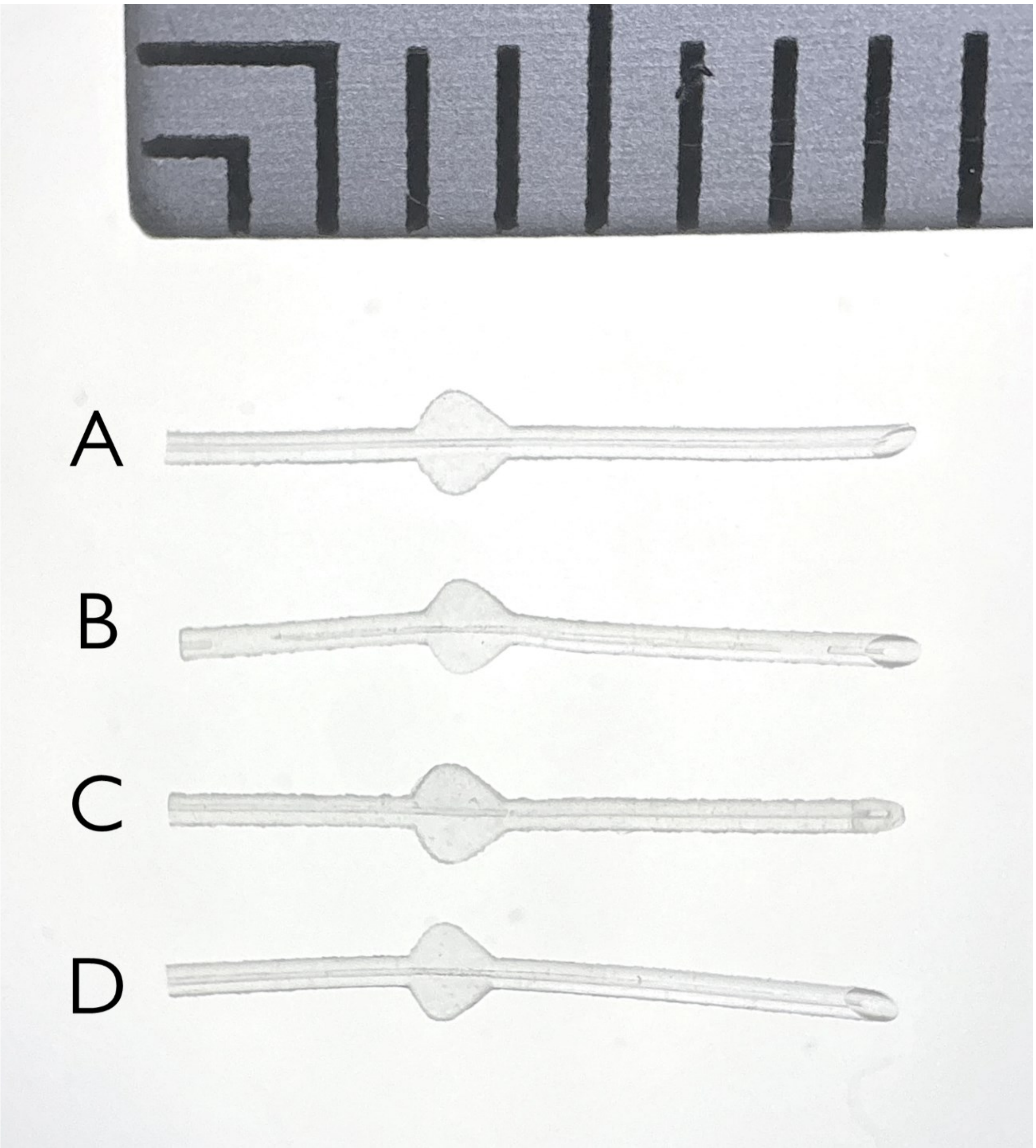

Online Resource 1. Comparative Photographs of MicroShunts without swelling.

Photographic comparison of MicroShunt illustrating no size changes. **A**, Unused MicroShunt (control). **B**, MicroShunt explanted from case 5. **C**, MicroShunt explanted from case 6. **D**, MicroShunt explanted from case 7.
