# Supplementary material for: Petrolatum-based ointment application induces swelling of the PRESERFLO Microshunt
Source: Graefes Arch Clin Exp Ophthalmol. 2026 Jan 13;264(5):1489–97. doi: 10.1007/s00417-025-07075-2 (PMC13091893; doi:10.1007/s00417-025-07075-2)
Supplement: Supplementary file 3 — (PDF 575 KB) [file 417_2025_7075_MOESM3_ESM.pdf]

Online Resource 4: Quantitative Analysis of Swollen MicroShunt caused by Ophthalmic Ointment using Proton nuclear magnetic resonance (^{1}H NMR) Spectroscopy

^{1}H NMR spectroscopy was performed to determine the composition of MicroShunt after immersing MicroShunt composed of poly(styrene-b-isobutylene-b-styrene [SIBS]) in 0.3% ofloxacin ophthalmic ointment for 24 hours and 3 months. The primary component of ophthalmic ointment is petrolatum, which is composed of alkanes. Therefore, "ophthalmic ointment" will be considered largely synonymous with "alkanes." Similarly, "MicroShunt" will be treated as synonymous with its constituent polymer, SIBS. Chemical shifts of the ^{1}H NMR spectrum are reported in parts per million (ppm) relative to tetramethylsilane (TMS) as an internal standard (δ = 0.00 ppm) in deuterated chloroform ($CDCl_3$).

1. Characterization of Components

Prior to analyzing the swollen MicroShunt, each constituent was characterized independently.

1.1. SIBS Polymer: The ^{1}H NMR spectrum of the pure SIBS polymer exhibited characteristic signals for aromatic protons from the styrene (S) blocks and aliphatic protons from the isobutylene (IB) blocks. By comparing the integrated areas of these signals, the molar ratio of the S and IB monomer units was determined to be 18:82. Using the molecular weights of the respective monomers (Styrene: 104 g/mol; Isobutylene: 56.1 g/mol), the weight ratio of the blocks (W_S: W_{IB}) was

calculated as

$W_S: W_{IB} = (18 \times 104): (82 \times 56.1) = 1: 2.40$

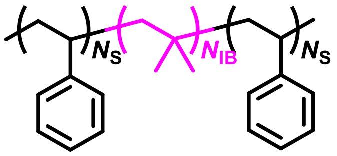

Chemical structure of the poly(styrene-b-isobutylene-b-styrene [SIBS]) copolymer.

N_s represents the degree of polymerization of the styrene blocks, and N_{IB} represents the degree of polymerization of the central isobutylene block.

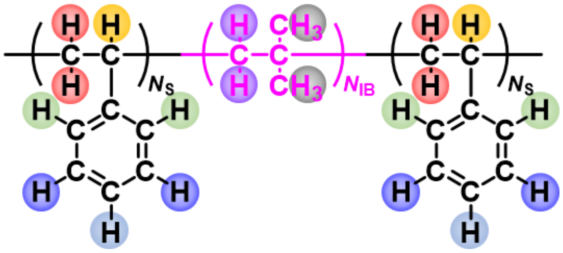

Schematic representation of the distinct proton environments in the SIBS polymer.

The protons are color-coded for direct correlation with their corresponding signals in the ^1H NMR spectrum shown in the ^1H NMR spectrum of the pure SIBS polymer.

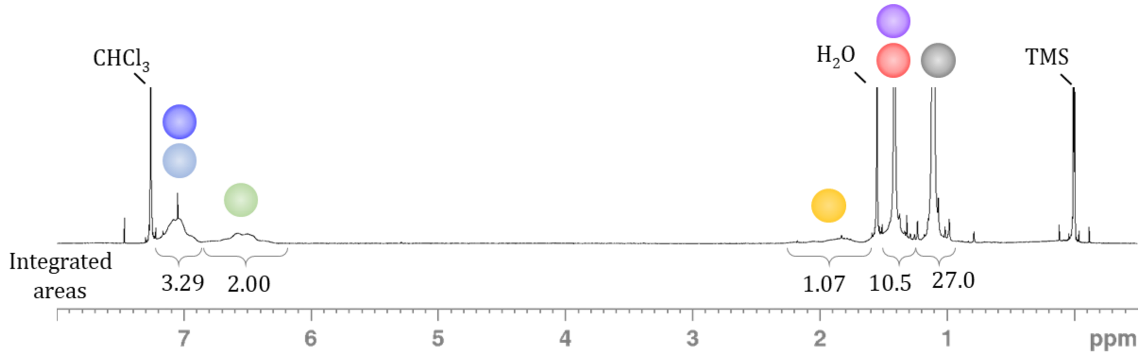

The ^1H NMR spectrum of the pure SIBS polymer.

The colored spheres above the signals correspond to the specific proton environments illustrated in the schematic of the distinct proton environments in the SIBS polymer. The signals in the aromatic region arise from the styrene blocks, whereas those in the aliphatic region arise from the isobutylene block. The integrated areas of these distinct signals are used to calculate the molar ratio of the styrene and isobutylene monomer units.

1.2. 0.3% Ofloxacin Ophthalmic Ointment: The ^1H NMR spectrum of the ophthalmic ointment confirmed its composition as a mixture of alkanes, showing signals corresponding to methyl ($-CH_3$), methylene ($-CH_2-$), and methine (>CH-) protons. The ratio of the number of methyl, methylene, and methine groups (n_{Methyl}: $n_{Methylene}$: $n_{Methine}$) was calculated by normalizing their integrated signal areas by their respective proton counts, yielding a ratio of 2:8.5:1.3. Subsequently, assuming the absence of quaternary carbons, this group ratio was used to estimate an

average empirical formula for the alkane mixture as $C_{11.8}H_{24.3}$, with a corresponding formula weight of 166 g/mol. However, this result does not reveal which alkanes are the ophthalmic ointment contains.

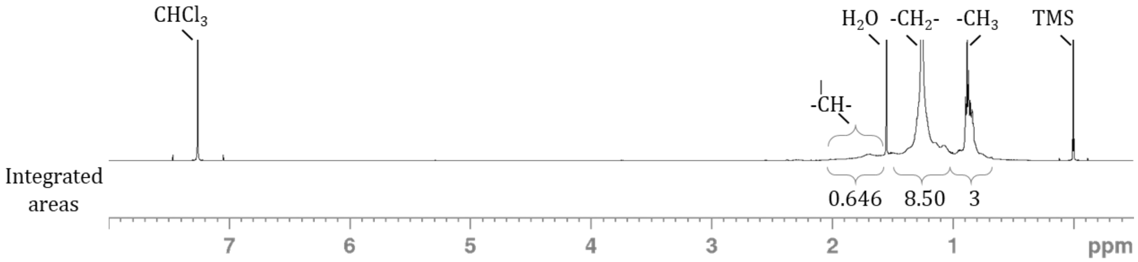

^{1}H NMR spectrum of the 0.3% ofloxacin ophthalmic ointment.

The characteristic signals for methyl (-CH_3), methylene (-CH_2-), and methine (-CH-) protons are observed in the aliphatic region. The integrated areas of these aliphatic signals were used to calculate the group ratio and estimate an average empirical formula for the alkane base.

2. Calibration of the Quantitative Method

To establish a robust correlation between NMR signal intensity and mass, a calibration standard was prepared by mixing a precisely known weight of pure polystyrene (S) with an equal weight of the ophthalmic ointment to create a 1:1 ratio. The ^{1}H NMR analysis of this mixture yielded a total integrated proton ratio of polystyrene to alkane (n_S: n_{Alkane})

of 1:1.84. This established a critical conversion factor, demonstrating that for this specific material pair, a measured proton ratio can be converted to a weight ratio using the following relationship:

$W_S : W_{Alkane} = n_S : (n_{Alkane} / 1.84)$

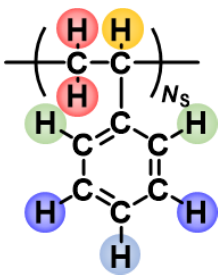

Schematic of the distinct proton environments in a styrene monomer unit.

The protons are color-coded for correlation with the corresponding signals in the ^1H NMR spectrum of the calibration mixture.

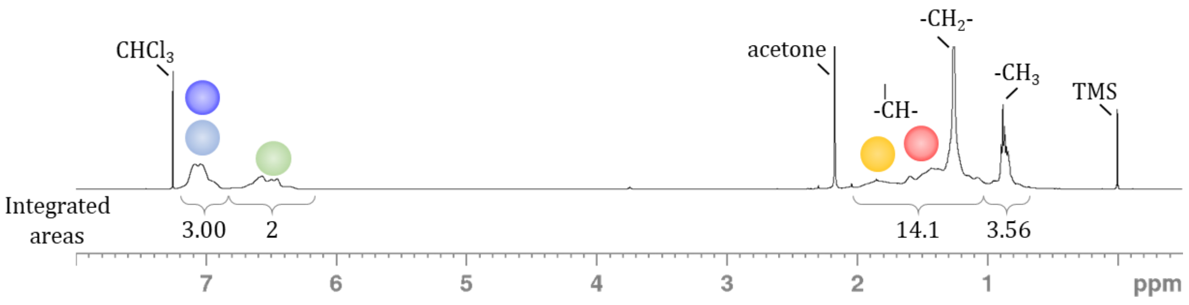

The ^1H NMR spectrum of the mixture of polystyrene in a known weight and ophthalmic ointment.

The spectrum displays a superposition of signals from the mixture of polystyrene in a

known weight and ophthalmic ointment and the weight ratio was 1:1. The colored spheres highlight the signals corresponding to the polystyrene protons, as illustrated in the schematic representation of the distinct proton environments in a styrene monomer unit. The total integrated area of these polystyrene signals was compared against the integrated area of the underlying signals from the alkanes in ophthalmic ointment to determine the proton ratio (n_S: n_{Alkane}).

---

3. Quantitative Analysis of the Swollen MicroShunt (First Method)

Two swollen MicroShunt samples, after immersion for 24 hours and 3 months respectively, were dissolved and analyzed by ^{1}H NMR. The final weight ratio of the SIBS polymer to the absorbed ophthalmic ointment (W_{SIBS}: W_{Alkane}) was determined for each sample using the calibration factor derived from the analysis of the 1:1 standard mixture.

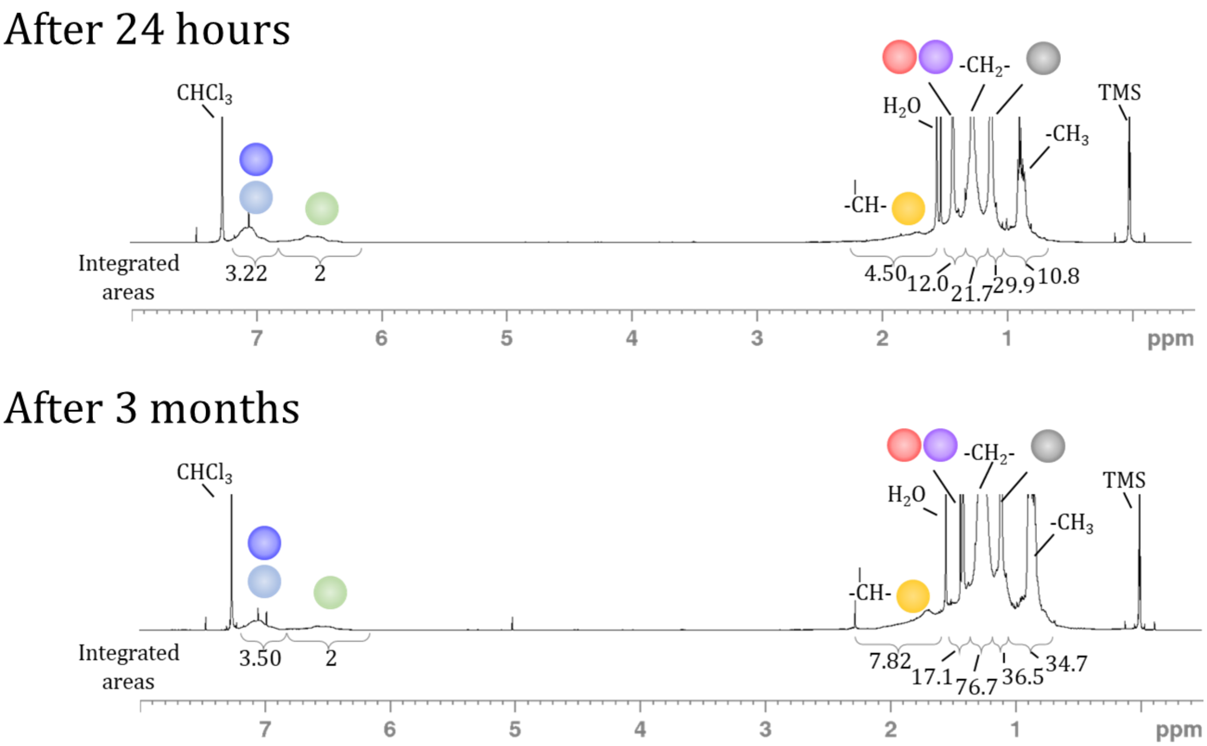

The ^{1}H NMR spectrum of the swollen MicroShunt after immersion for 24 hours and 3 months.

The spectrum displays a superposition of signals from both the SIBS polymer and the absorbed ophthalmic ointment after (top) 24 hours and (bottom) 3 months. The colored spheres highlight the characteristic signals of the SIBS component, with colors corresponding to the proton environments detailed in the schematic representation of the distinct proton environments in the SIBS polymer. The aromatic signals are exclusive to the styrene blocks of SIBS. The aliphatic region contains signals from the isobutylene blocks superimposed upon the significantly larger signals from the absorbed ophthalmic ointment. The marked increase in the relative integrated area of the aliphatic signals in the three-month sample represents the greater mass of absorbed ophthalmic ointment, which forms the basis for the quantitative analysis.

For the 24-hour sample, analysis of the NMR spectrum yielded a ratio of the total integrated protons from the styrene (S) component to those from the absorbed ointment (n_S: n_{Alkane}) of 1:5.04. The final weight ratio was calculated in a two-step process:

1. The weight ratio of the styrene portion of SIBS to the ophthalmic ointment (W_S: W_{Alkane}) was calculated using the proton ratio and the calibration factor:

   W_S: W_{Alkane} = n_S: (n_{Alkane} / 1.84) = 1: (5.04 / 1.84) = 1:2.74

2. Using the known internal weight ratio of the SIBS polymer (W_S: W_{IB} = 1:2.40), the weight ratio of the total SIBS polymer to the ophthalmic ointment (W_{SIBS}: W_{Alkane}) was determined:

   W_{SIBS}: W_{Alkane} = (W_S + W_{IB}): W_{Alkane} = (1 + 2.40): 2.74 = 3.40:2.74

   This simplifies to a final weight ratio of 1:0.805, approximately 55:45.

Analysis of the NMR spectrum for the three-month sample yielded a ratio of the total integrated protons from the styrene (S) component to those from the absorbed ointment (n_S: n_{Alkane}) of 1:16.8. The final weight ratio was calculated in a two-step process:

1. The weight ratio of the styrene portion to the ointment was calculated:

   W_S: W_{Alkane} = n_S: (n_{Alkane} / 1.84) = 1: (16.8 / 1.84) = 1:9.13

2. The total weight ratio of SIBS to the ointment was then determined:

W_{SIBS}: W_{Alkane} = (W_S + W_{IB}): W_{Alkane} = (1 + 2.40): 9.13 = 3.40:9.13

This simplifies to a final weight ratio of 1:2.69, approximately 27:73.

4. Validation using an Assumed Ointment Molecular Structure (Second Method)

To validate the primary results, a secondary analysis was performed using a different model for the ophthalmic ointment. Assuming the absence of quaternary carbons and a structural multiplier of (*x = 2)* based on the physical properties of the ophthalmic ointment, the initial empirical formula of $C_{11.8}H_{24.3}$ was refined to an average molecular formula of $C_{23.6}H_{48.6}$ (molecular weight of 332 g/mol) for the validation. This model assumes that the alkanes are predominantly linear or lightly branched without quaternary carbons.

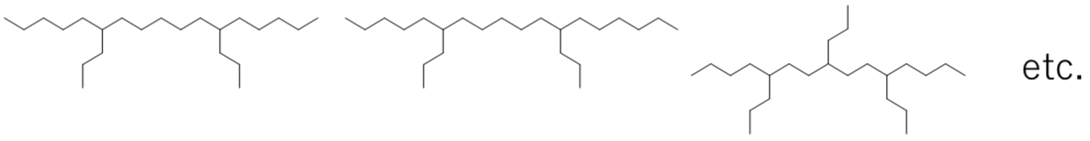

Representative alkane structures postulated for the ophthalmic ointment in the validation.

Schematics of possible alkane structures present in the ophthalmic ointment were consistent with the absence of quaternary carbons and suggested a range of chain lengths and branching patterns. The "etc." indicates that these are illustrative examples and not exhaustive.

For the 24-hour sample, starting from the measured proton ratio (n_S: n_{Alkane} = 1:5.04):

1. The proton ratio was converted to a molar ratio of styrene monomer units (n_S) to average alkane molecules (n_{Alkane}) by dividing by the number of protons in each unit (8 for a styrene monomer, 48.6 for the model alkane):

   n_S: n_{Alkane} = (1/8) : (5.04/48.6) = 1:0.830
2. This molar ratio was converted to a weight ratio (W_S:W_{Alkane}) using the molecular weights (104 g/mol for styrene, 332 g/mol for the model alkane):

   W_S: W_{Alkane} = (1 × 104): (0.830 × 332) = 1:2.65
3. Finally, this was combined with the SIBS block ratio to find the total weight ratio:

   W_{SIBS}: W_{Alkane} = (1 + 2.40): 2.65 = 3.40:2.65

   This simplifies to a final weight ratio of 1:0.779, approximately 56:44.

For the three-month sample, starting from the measured proton ratio (n_S: n_{Alkane} = 1:16.8):

1. The proton ratio was converted to a molar ratio:

   n_S: n_{Alkane} = (1 / 8) : (16.8 / 48.6) = 1:2.77

2. This was converted to a weight ratio using molecular weights:

   W_S: W_{Alkane} = (1 × 104): (2.77 × 332) = 1:8.83

3. Finally, this was combined with the SIBS block ratio to find the total weight ratio:

   W_{SIBS}: W_{Alkane} = (1 + 2.40): 8.83 = 3.40:8.83

This simplifies to a final weight ratio of 1:2.60, approximately 28:72.

5. Conclusion

The quantitative ^{1}H NMR analysis determined the weight ratio of the MicroShunt to the absorbed ophthalmic ointment after 24 hours and 3 months of swelling to be 55:45 and 27:73. These results were corroborated by a secondary, independent analysis based on an assumed molecular structure for the ointment, which yielded ratios of 56:44 and 28:72.
